# Personal Protective Equipment and Risk for Avian Influenza (H7N3)

**DOI:** 10.3201/eid1501.070660

**Published:** 2009-01

**Authors:** Oliver Morgan, Mirjam Kuhne, Pat Nair, Neville Q. Verlander, Richard Preece, Marianne McDougal, Maria Zambon, Mark Reacher

**Affiliations:** Health Protection Agency, Cambridge, UK (O. Morgan, P. Nair, M. Reacher); Health Protection Agency, London, UK (M. Kuhne, N.Q. Verlander, M. Zambon); Atos Origin, London (R. Preece); Department for Environment, Food and Rural Affairs, London (M. McDougal); 1 Current affiliation: Centers for Disease Control and Prevention, Atlanta, Georgia, USA.

**Keywords:** Avian influenza, disease outbreaks, poultry, dispatch

## Abstract

An outbreak of avian influenza (H7N3) among poultry resulted in laboratory-confirmed disease in 1 of 103 exposed persons. Incomplete use of personal protective equipment (PPE) was associated with conjunctivitis and influenza-like symptoms. Rigorous use of PPE by persons managing avian influenza outbreaks may reduce exposure to potentially hazardous infected poultry materials.

In April 2006, an outbreak of avian influenza occurred on 3 poultry farms in Norfolk, England (*1*). Reverse transcription–PCR (RT-PCR) of poultry blood samples and cloacal swabs detected low-pathogenic avian influenza (H7N3) on 1 farm, and veterinary investigation confirmed influenza subtype H7N3 on the 2 adjacent farms. Surveillance and protection zones were established around all infected premises, and all birds were culled. Persons who had been exposed were offered oseltamivir prophylaxis; those with influenza symptoms were offered oseltamivir treatment and influenza vaccination. All persons at risk were orally instructed to wear personal protective equipment (PPE).

## The Study

We conducted a retrospective cohort study of all persons who had been potentially exposed to infectious material by handling live and dead poultry, poultry products, or litter derived from infected premises. Our objective was to measure associations between potential exposure to infectious material, completeness of use of PPE, and taking and timing of oseltamivir prophylaxis with having symptoms consistent with or confirmed as resulting from influenza virus A (H7N3) infection. We pretested and then administered a questionnaire by telephone after poultry culling ended (median 66 days, range 60–143 days). For persons who did not respond to the questionnaire (n = 39), we extracted data recorded in the outbreak records to describe their activities in relation to the outbreak, their use of oseltamivir prophylaxis, and their seasonal influenza vaccine status. Only persons who were interviewed and completed the questionnaire (n = 103) were included in the statistical analysis. Persons were invited to provide an acute-phase blood sample during the outbreak and a convalescent-phase sample 28 days after their last potential exposure. Exceptions were those at low risk; e.g., incinerator workers and lorry drivers.

Possible case-patients were those who reported conjunctivitis or influenza-like symptoms (>1 of the following: fever, sore throat, cough, shortness of breath, body/muscle pain, runny nose) in the 7 days after last potential exposure. Confirmed case-patients were those for whom virus was detected by culture and RT-PCR of material from the conjunctiva or respiratory tract and/or confirmed by serologic testing. Influenza virus (H7N3) from the conjunctiva of the index case-patient was prepared by growth in embryonated eggs. Serum samples were screened by using microneutralization (MN) and hemagglutination inhibition (HI) tests (*2**,**3*). We defined MN >20 as evidence of seroreactivity. When either test gave a positive result, we performed confirmatory Western blot analysis, using purified influenza (H7N3) virus (*4*).

We calculated odds ratios (ORs), 95% confidence intervals (CIs), and p values for being a possible or confirmed case-patient. Independent variables are shown in the Technical Appendix, Table A. All risk factors with p<0.2 in the single-variable analysis were initially included in a logistic regression model and then removed, least significant first, until all had p<0.1. Confounding variables (those that caused >10% change in the ORs of covariates) were retained regardless of p value.

In total, 142 persons were potentially exposed. Questionnaires were completed for 103 (73%) persons (21 could not be contacted, 10 declined, 7 had no contact information, and 1 questionnaire was lost). Characteristics, potential exposures, and preventive measures differed little between persons who did or did not complete the questionnaire (Table 1). Of 46 persons who reported symptoms, 19 reported conjunctivitis with influenza-like symptoms and 27 reported influenza-like symptoms only. PPE reported as “always used" were protective coveralls (81%), protective footwear (82%), disposable gloves (67%), face-fitted mask (51%), other mask (24%), and protective goggles (19%) (Technical Appendix, Table B).

**Table 1:** Characteristics and preventive measures taken by all persons potentially exposed to influenza A virus A (H7N3)–infected materials

Characteristic	Completed questionnaire (n = 103), no. (%)*	Did not complete questionnaire (n = 39), no. (%)†
Male gender	81 (79)	34 (87)
Oseltamivir prophylaxis		
Yes	98 (95)	30 (77)
No	5 (5)	6 (15)
Unknown		3 (8)
Seasonal influenza vaccine		
Received before outbreak	5 (5)	1 (3)
Received during outbreak	12 (12)	1 (3)
Received before and during outbreak	66 (64)	29 (74)
Not received	8 (8)	2 (5)
Unknown	12 (12)	6 (15)
Activities on infected premises‡	65 (63)	22 (56)
Any activity with potentially high exposure§	62	6
Catching poultry	39	4
Culling poultry	24	7
Inspecting or collecting biological/environmental samples	21	2
Loading dead poultry for transport	32	0
Disinfecting and cleaning	17	2
Activities off infected premises	38 (37)	12 (31)
Running incinerator	16	8
Transporting dead poultry	10	0
Testing biological/environmental samples	4	1
Other	8	3
Activities unknown	0	5 (13)
Use of personal protective equipment¶		
Complete	56 (54)	–
Incomplete	47 (46)	–
Exposure to poultry during 6 mo before outbreak		
Never	20 (19)	–
Occasional	34 (33)	–
Frequent	46 (45)	–
Unknown	3 (3)	–
Symptoms reported 7 d postexposure		
Conjunctivitis only	0	–
Influenza-like symptoms only	27 (26)	–
Conjunctivitis and influenza-like symptoms	19 (18)	–
Influenza-like symptoms#	46 (45)	–
Body/muscle pain	23	–
Sore throat	22	–
Runny nose	16	–
Cough	15	–
Shortness of breath	8	–
Fever (subjective, not measured)	5	–

*Median age (range) 40 (15–64) y. †Median age (range) 41 (19–74) y. ‡For the 39 persons who did not respond to the study questionnaire, we used activities recorded in the outbreak records. Some persons had >1 exposure on site. §High exposure includes >1 of the following activities: entering poultry sheds, coming within 1 m of live poultry, handling live or dead poultry, contact with chicken litter or feathers, and handling eggs or egg products. ¶Complete use of personal protective equipment defined as always using gloves, coveralls, footwear, face-fitted N95 respirator, or other mask (unspecified), and goggles. #Reported by the 27 patients with influenza-like symptoms only and the 19 with conjunctivitis and influenza-like symptoms.

Fifty-six (54%) persons reported complete use of PPE. Single-variable analysis indicated that working on an infected premise (OR 2.76, 95% CI 1.17–6.50) was significantly associated with being a possible or confirmed case-patient (Technical Appendix, Table A). Higher levels of exposure to potentially infected poultry (OR 2. 20, 95% CI 0.96–5.04) and only partial use compared with full use of PPE (OR 2.16, 95% CI 0.97–4.83) were also associated with being a possible or confirmed case-patient, but 95% CIs were <1.0. Characteristics not associated with being a possible or confirmed case-patient were age >30 years; male sex; being a Department for Environment, Food and Rural Affairs employee; smoking; having had a prior influenza vaccination; timing of starting oseltamivir prophylaxis; and exposure to potentially infected poultry in the preceding months. Multivariable analysis showed the association with being a possible or confirmed case-patient to be statistically significant for incomplete use of PPE and weakly significant for working on an infected premise (Table 2).

**Table 2:** Multivariable analysis of factors associated with possible or confirmed cases*

Factor	Odds ratio	95% CI	p value
Defra employee			
No	1.00		
Yes	2.07	0.72–5.94	0.17
Working on an infected premise			
No	1.00		
Yes	7.53	0.68–83.41	0.064
Potential exposure level			
Low	1.00		
High	0.26	0.02–2.75	0.22
Use of personal protective equipment			
Complete	1.00		
Incomplete	3.26	1.22–8.73	0.015
Use of oseltamivir relative to first potential exposure			
Before	1.00		
On the same day	2.19	0.84–5.71	0.27
After	1.33	0.29–6.09	

*Based on 96 questionnaires with complete information. CI, confidence interval; Defra, Department for Environment, Food and Rural Affairs.

Serum samples were available from 91 persons: 33 acute- and convalescent-phase pairs, 49 acute-phase samples, and 9 convalescent-phase samples. Only the serum from the index case-patient showed reactivity in both the MN (titer 40) and HI (titer 32) tests and also showed reactivity in Western blot. No acute-phase sample from this person was available. All other acute- and convalescent-phase samples were negative in both tests. During the outbreak, eye, nose, and throat swabs were taken from 14 persons (1–8 days after symptom onset); 10 reported influenza-like symptoms (2 without eye involvement), 2 reported no symptoms, and 2 had no clinical information available. Comprehensive molecular diagnostic tests for common human viral respiratory pathogens (enteroviruses, rhinoviruses, adenoviruses, respiratory syncytial viruses, parainfluenza viruses) were also performed and did not provide evidence of alternative causes of infection. A vaccine strain of avian paramyxovirus (Newcastle disease virus) was recovered from 1 person with conjunctivitis, which suggests that at least 1 case of conjunctivitis was caused by avian paramyxovirus. Serologic testing for seasonal influenza infection (HI tests on all paired serum samples) did not indicate any recent human infections.

Our study had a number of limitations. Because workers were interviewed a minimum of 2 months after the outbreak, they may not have accurately recalled their exposures. In addition, we relied on self-reported data. Difficulties recalling symptoms were less likely as we actively followed up persons for 7 days after last exposure. In the absence of a control group, such as farmers from noninfected premises, whether the incidence of influenza-like illness and conjunctivitis in this cohort was different is unclear, although during the outbreak, influenza activity in the general population was low and no isolates of seasonal influenza were reported. We did not measure dust exposure as an alternative explanation for conjunctivitis in some or all persons, apart from the index case-patient who reported this symptom. The results from laboratory testing were limited because convalescent-phase serum was not available from all persons who reported influenza-like illness. However, a wide range of molecular diagnostic tests for human viral pathogens were performed on samples from persons who were not well at the time of the outbreak. Because the kinetics of appearance and disappearance of human antibodies to avian influenza are poorly understood, timing of the collection of samples may not have been optimal in this outbreak and we may have missed the opportunity to diagnose some infections. Moreover, because serologic tests for influenza virus A (H7N3) may not correlate well with infection (*5*), we could not rule out influenza A virus (H7N3) infection among symptomatic persons, even in the presence of convalescent-phase serum that was negative for H7.

## Conclusions

Strict compliance with PPE use should be reinforced when outbreaks of avian influenza among poultry are being managed, as recommended in current guidance from the United Kingdom (*6*) and the European Centre for Disease Prevention and Control (*7*). Compliance tends to be suboptimal (*8*), possibly because of low risk perception among poultry workers (*9*). Understanding what obstacles prevent workers from wearing complete PPE is needed. Our study suggests that rigorous use of PPE by persons managing avian influenza outbreaks reduces influenza-like symptoms and conjunctivitis and potentially hazardous exposure to infected poultry materials.

## Supplementary Material

Technical Appendix Personal Protective Equipment and Risk for Avian Influenza (H7N3)
