# Supplementary material for: Personal Protective Equipment and Risk for Avian Influenza (H7N3)
Source: Emerg Infect Dis. 2009 Jan;15(1):59–62. doi: 10.3201/eid1501.070660 (PMC2662632; doi:10.3201/eid1501.070660)
Supplement: Technical Appendix — Personal Protective Equipment and Risk for Avian Influenza (H7N3) [file 07-0660_Techapp-s1.pdf]

Personal Protective Equipment and Risk for Avian Influenza (H7N3)

Technical Appendix

Table A. Single-variable analysis of risk factors for reporting influenza-like symptoms and conjunctivitis*

Factor	Symptoms† Present	Symptoms† Absent	Odds ratio	95% CI	p value
Age, y					
≥30	33	46	1.00		
<30	13	10	1.81	0.71–4.63	0.21
Sex					
Female	35	46	1.00		
Male	11	10	1.45	0.55–3.79	0.45
Defra employee					
No	28	41	1.00		
Yes	18	15	1.76	0.76–4.06	0.19
Working on any infected premises					
No	11	26	1.00		
Yes	35	30	2.76	1.17–6.50	0.017
Exposure level					
Low	13	26	1.00		
High‡	33	30	2.20	0.96–5.04	0.059
Smoker (n = 100)					
No	28	39	1.00		
Yes	17	16	1.48	0.64–3.42	0.36
Influenza vaccination before outbreak (n = 91)					
No	35	37	1.00		
Yes	9	10	0.95	0.35–2.62	0.92
Use of personal protective equipment					
Complete§	16	30	1.00		
Incomplete¶	30	26	2.16	0.97–4.83	0.057
Use of oseltamivir relative to first potential exposure (n = 96)					
Before	23	33	1.00		
On the same day	19	11	2.48	0.99–6.18	0.12
After	4	6	1.43	0.33–6.33	
Exposed to poultry in past 6 months (n = 99)					
Never	7	13	1.00		
Occasionally	18	16	2.09	0.67–6.53	0.43
Frequently	20	25	1.49	0.50–4.42	

*N = 102 unless otherwise indicated; data on symptoms were missing for 1 person, resulting in 102 persons with complete data. CI, confidence interval; Defra, Department for Environment, Food and Rural Affairs.
†Symptom onset was in the 7 days after most recent exposure.
‡Entering poultry sheds; coming within 1 m of live poultry, handling live or dead poultry, contact with chicken litter or feathers, and handling eggs or egg products.
§Always used gloves, coveralls, footwear, face-fitted N95 respirator, or other mask (unspecified) and goggles.
¶≥1 item used sometimes or never.

Table B. Self-reported use of personal protective equipment items

Equipment	No. (%) Never	Sometimes	Always	Missing
Disposable gloves	20 (19)	12 (12)	69 (67)	2 (2)
Protective coveralls	13 (13)	5 (5)	83 (81)	2 (2)
Protective footwear	15 (15)	1 (1)	84 (82)	3 (3)
Face-fitted mask	32 (31)	9 (9)	53 (51)	9 (9)
Other mask	48 (47)	14 (14)	25 (24)	16 (16)
Protective goggles	67 (65)	8 (8)	20 (19)	8 (8)
